# Indonesian primary care physicians profile in 2011: Did practicing hours and conversion program for family medicine differentiate their services and continuing medical education activities?

**DOI:** 10.1186/s12930-014-0016-x

**Published:** 2014-12-20

**Authors:** Indah S Widyahening, Daniel M Thuraiappah, Tin Myo Han, Dhanasari Vidiawati

**Affiliations:** Department of Community Medicine, Faculty of Medicine Universitas Indonesia, Jl. Pegangsaan Timur 16, Jakarta, 10430 Indonesia; Academy of Family Physicians of Malaysia and Family Medicine Department, MAHSA University College, Kuala Lumpur, Malaysia; Myanmar Medical Association- General Practitioners’ Society. Medical Statistics Unit, Faculty of Dentistry, International Islamic University, Kuala Lumpur, Malaysia

**Keywords:** Primary health care, General practice, Family practice, Health service survey, Indonesia

## Abstract

**Background:**

In Indonesia, Family Medicine as a discipline is being developed through short courses since 12 years ago. A conversion program to become Family Physicians has been introduced recently. Among the 70,000 primary care physicians there are variety of practitioners, from new interns who start general practice to senior general practitioners. This study aims to describe the current Indonesian Primary Care Physicians (PCPs) profile which includes services provided and facilities as well as comparing the profile according to participation in the conversion program and practice hours.

**Methods:**

A survey was carried out by using pre-tested, semi-structured and self-administered questionnaire among Indonesian primary care physicians (PCPs) who attended ASEAN Regional Primary Care Conference in Jakarta, November 2011. The survey elicited information regarding their practice environment, services provided, equipment, investigations provided, procedures, facilities and continuing medical education (CME) activities.

**Results:**

Out of 240 PCPs participated, 65.4% (157/240) of them were family physicians and 67.1% (161/240) of them were full time practitioners (practice > 30 hours per week). Services like body mass index (BMI) measurement, substance abuse program, respiratory function test, mental health assessment, and cardiovascular assessment were provided by less than 50% of the PCPs as well as some investigations like electrocardiograph (ECG), proctoscopy, ultrasound, visual examination and funduscopy. Family Physicians significantly provided more house call services (77% vs 63%; p = 0.01), than those who are not. No other significant difference was found in the practice of the family physicians compare to non-family physicians.

**Conclusions:**

The Indonesian PCPs were lacking in the provision of some particular medical procedures, management and follows up of acute and chronic conditions, and preventive medicine and health education. Improvement of primary health care has been seen globally as necessary effort in health systems reform and this information could provide guidance toward the efforts to improve the quality of primary care physicians in Indonesia.

## Background

Primary care is one level of health system which provides first point of care to the population and has easy access, low cost, continuous, coordinated and comprehensive care as its core attributes [1]. General practitioners/family physicians hold a central role in provision of healthcare services in most countries even though there are variations with regard to the levels of training, organization and service deliveries [2].

Different surveys to describe the profile of primary care practice has been conducted in several countries [3-6]. However, none of these surveys described the primary care practice in South East Asian countries. Indonesia is the biggest country with the largest population within the South East Asian countries. The number of general practitioners (GP) in Indonesia is around 70,000 while the specialist is 16,000. Currently, every medical school graduates in Indonesia is prepared to practice as GP after they complete a one year internship program. Family medicine is not yet recognized as a specialty. Other neighboring countries such as Malaysia, Singapore and the Philippines, have general practice vocational training program (3–4 year), commencing after a basic medical education degree [7]. To make the Indonesian GP’s qualification equal with other South East Asian countries, a structured post-graduate training program is currently being developed. As part of the preliminary process, a conversion program is being conducted to accredit GPs who already implement certain level of family medicine approach in their clinical practice. The conversion program is intended for GPs who have been in practice for at least 5 years and undergo an assessment of competence for the GPs who want to improve their status to family practitioners [8]. They have to complete a form which serves as record of their past and current medical practice and professional activities. After the conversion, they become the member of the Indonesian Association of Family Physicians (PDKI) with status as Family Physicians and need to participate in continuing professional development program on a regular basis.

This survey is part of a surveys conducted in four countries (Indonesia, Malaysia, Myanmar and the Philippines) to assess the current Primary Care Physicians/General Practitioners profile and their CME activities. In this report we only describe the Indonesian data and aim to compare the profile of those participated in the conversion program with those who do not participated as well as comparing based on the practice hours.

## Methods

A cross-sectional descriptive and analytic study was carried out by using pre-tested, semi-structured and self-administered questionnaire among Indonesian primary care physicians (PCPs) who attended the 2nd ASEAN Regional Primary Care Conference in Jakarta, November 2011. The survey form is modified from the Malaysian Quality Improvement Program which has detailed surveys on structure, clinical processes and clinical outcomes [9]. The modified version has been used to profile practicing doctors in Malaysia. The survey elicited information which reflect four areas of service provision provided by primary care: 1) as the doctor of first contact in health-related matters, 2) in minor surgical and investigative procedure, 3) in the management and follow-up of a broad range of acute and chronic diseases, and 4) in preventive medicine [5,6]. The items in the questionnaire were classified in to practice environment, services provided, equipment, investigations provided, procedures, facilities and continuing medical education (CME) activities in accordance with the Malaysian Private Healthcare Facilities and Services Act Regulation 2008 which is then adjusted to the Standard of Practice of the Indonesian Family Physicians.

A pilot test was done and modification of the questionnaires was done in accordance with the finding of the pilot test. Reliability of over all 46 items of the data collecting tool (Cronbach’s alpha) was 0.804. Indonesian PCPs who practiced more than 30 hours per week are defined as full time practitioners while those who practiced less than 30 hours per week are classified as part-time practitioners. The PCPs were also classified into family physicians who passed the conversion program and non- family physicians who have not yet attained the conversion program.

The questionnaire was put in the delegates pack together with an information leaflet on consent for survey participation. Returning of the self-completed questionnaire by responders was taken as their consent. The Health Research Ethics Committee of the Faculty of Medicine Universitas Indonesia reviewed and approved the study.

A cross analysis using chi-square or fisher test (as appropriate) was done to find out the association between practice hours, conversion program and their practices. All analyses were performed using the SPSS 11.0 (SPSS Inc., Chicago, IL).

## Results

Total 240 Indonesian PCPs participated in the study; 144 of them (60%) were female. Most of them (175/240, 73%) practice in Java (the most populated island in Indonesia) or in the provincial capital cities of Indonesia. Out of 240, 65.4% (157/240) of them were Family Physicians and 67.1% (161/240) of them were full time practitioners. Majority (150/157, 95%) of the family physicians practiced more than 5 years and 83% (134/161) of full-time PCPs were the main practitioners in the clinic.

The Family Physicians significantly provided more house call services (77% vs 63%; p = 0.01), than non-family physicians. Certain aspects were found more in family physicians such as dispensing medicine in clinic, certifying workers for fitness, women’s health services, family planning services, providing substance abuse program, cardiovascular assessment, prescribing herbal medicine to some patients, medical nutrition therapy, satisfactory with medical equipment they have, doing urine examination, blood glucose test, visual examination, fundoscopy, soft tissue infiltration, cosmetic surgery, and keeping medical record; but these variations were not statistically significant (Tables 1 and 2).

**Table 1:** **Practice environment, services, investigation and procedures provided by primary care physicians (PCPs) in Indonesia (N = 240); comparison based on the participation in Family Medicine conversion program and practice hours**

	**Overall**		**PCPs classification**					**Practice hours per week**				
			Family physicians* (N = 157)		**Non-Family Physicians (N = 83)**		p***	**>30 hours (N = 161)**		**<30 hours (N = 79)**		p***
	**n**	**%**	**n**	**%**	**n**	**%**		**n**	**%**	**n**	**%**	
**Practice environment**												
Full time practice**	161	67	102	65	59	71	0.34	-	-	-	-	
Practice > 5 years	221	92	150	96	71	86	<0.01	148	92	73	92	0.9
Main practitioners in the clinic	185	77	123	78	62	75	0.42	134	83	51	66	<0.01
**Services**												
Facilities for emergency care	191	80	129	82	62	75	0.07	136	85	55	70	0.01
House call	173	72	121	77	52	63	0.01	116	72	57	72	0.46
Dispensing Medicine in clinic	191	80	128	82	63	76	0.35	130	81	61	77	0.81
Immunization	170	71	110	70	60	72	0.88	121	75	49	62	0.1
Measuring BMI	110	46	72	46	38	46	0.92	82	51	28	35	0.06
Certifying workers for fitness	151	63	103	66	48	58	0.12	105	65	46	58	0.15
Women/reproductive health	169	70	114	73	55	66	0.93	117	73	52	66	0.37
Family planning services	198	83	135	86	63	76	0.05	133	83	65	82	0.88
Substance abuse program	89	37	59	38	30	36	0.61	67	42	22	28	0.04
Respiratory function test	78	33	47	30	31	37	0.32	56	35	22	28	0.51
Mental Health assessment	129	54	83	53	46	56	0.73	92	57	37	47	0.13
Cardiovascular assessment	127	53	85	54	42	51	0.36	91	57	36	46	0.16
Treadmill assessment	52	22	31	20	21	25	0.29	34	21	18	23	0.9
Prescribe herbal medicine	101	42	69	44	32	39	0.42	70	44	31	39	0.53
Medical nutrition therapy	158	66	108	69	50	60	0.08	108	67	50	63	0.76
**Equipment**												
Satisfactory with medical equipment they have	144	60	99	63	45	54	0.1	103	64	41	52	0.2
**Investigation**												
Urine examination	170	71	114	73	56	68	0.41	121	75	49	62	0.04
Blood glucose test	206	86	136	87	70	84	0.63	145	90	61	77	<0.01
ECGs	81	34	53	34	28	34	1	63	39	18	23	0.01
Proctoscopy	39	16	25	16	14	17	0.58	29	18	10	13	0.51
Ultrasound	68	28	44	28	24	29	0.91	46	29	22	28	0.88
Visual (visus) examination	138	58	95	61	43	52	0.2	97	60	41	52	0.22
Funduscopy	89	37	59	38	30	36	0.61	59	37	30	38	0.22
**Procedures**												
Minor surgery	204	85	132	84	72	87	0.7	143	89	61	77	0.03
Soft tissue infiltration	101	42	67	43	34	41	0.79	76	47	25	32	0.02
Acupuncture	49	20	25	16	24	29	0.02	33	21	16	20	0.97
Hypnotherapy	24	10	11	7	13	16	0.03	14	9	10	13	0.34
Cosmetic Surgery	35	15	24	15	11	13	0.7	20	12	15	19	0.3

Legend:

*Family physicians are those passed the conversion program by the Indonesian Association of Family Physicians.

** Full-time practice is practice more than 30 hours per week

***p is calculated with chi-square or fisher test as appropriate.

**Table 2:** **Clinic facilities and continuing medical education activities of primary care physicians in Indonesia (N = 240); comparison based on the participation in Family Medicine conversion program and practice hours**

	**Overall**		**PCPs classification**					**Practice hours per week**				
			Family physicians* (n = 157)		**Non-family Physicians (n = 83)**		p**	**>30 hours (n = 161)**		**<30 hours (n = 79)**		p**
	**n**	**%**	**n**	**%**	**n**	**%**		**n**	**%**	**n**	**%**	
**Facilities**												
Medical record	222	93	147	94	75	90	0.42	147	91	75	95	0.54
Patients’ Register	224	93	150	96	74	89	0.06	152	94	72	91	0.34
Separated register for chronic disease	152	63	94	60	58	70	0.26	110	68	42	53	0.04
Locking cupboard for dangerous drugs	158	66	101	64	57	69	0.51	107	67	51	65	0.85
Having a computer/laptop	222	93	144	92	78	94	0.53	150	93	72	91	0.58
Electronic Medical Record	119	50	71	45	48	58	0.06	87	54	32	41	0.04
Internet access	207	86	131	83	76	92	0.2	140	87	67	85	0.35
**Continuing medical education for GPs**												
Post Graduate Qualification	38	16	30	19	8	10	0.06	86	53	29	37	0.05
Short courses in Family Medicine	171	71	129	82	42-	51	<0.01	114	71	57	72	0.82
Conversion program	157	65	-	-	-	-	-	102	63	55	70	0.34
Reading > one journal per year	214	89	143	91	71	86	0.31	142	88	72	91	0.76
Attending > one ward round per year	149	62	101	64	48	58	0.61	103	64	46	58	0.54
Attending > five talk or lectures per year	144	60	90	57	54	65	0.35	102	63	42	53	0.27
Attending one workshop/symposium & conference per year	219	91	143	91	76	92	0.93	148	92	71	90	0.54
Attending a full course in the last five years	205	85	138	88	67	81	0.24	136	85	69	87	0.46
Literature search to answer patients’ problem (>1x /month)	207	86	136	87	71	86	0.97	142	88	65	82	0.12
Participated in Quality Improvement Program	195	81	133	85	62	75	0.17	134	83	61	77	0.11

Legend:

*Family physicians are those passed the conversion program by the Indonesian Association of Family Physicians.

**p is calculated with chi-square or fisher test as appropriate.

Fewer family physicians provide acupuncture (16% vs 29%; p = 0.02) and hypnotherapy (7% vs 16%; p = 0.03) compare to non-family physicians.

There was significant differences between full-time practitioners and part-time practitioners with reference to emergency care services (85%. vs 70% -p = 0.01), in providing substance abuse program (42% vs 28%; p = 0.04), doing urine examination (75% vs 62%; p = 0.04), blood glucose test (90% vs 77%; p < 0.01) and ECG (39% vs 23%; p = 0.01) at clinic, providing minor surgery (89% vs 77%; p = 0.03) and soft-tissue infiltration (47% vs 32%; p = 0.02); and keeping registers for chronic disease (68% vs 53%; 0.04) and electronic medical records (54% vs 41%; P = 0.04).

Regarding continuing medical education for general practitioners, no statistically significant different was found between the family physicians vs non-family physicians and the full-time vs part-time practitioners (Table 2).

## Discussion

This study demonstrates the variety of skills and services provided by some practitioners in order to examine whether any or all of the services which are essential in an Indonesian environment were provided. Fifty percent or less of the PCPs provides body mass index (BMI) measurement and cardiovascular assessment as well as providing substance abuse program and mental health assessment. Investigative procedures such as respiratory function test, electrocardiography, ultrasonography, visual examination with an ophthalmoscope and even proctoscopy are also low among the PCPs.

The range of services provided by primary care varies considerably from country to country. Boerma, et al. found that primary care physicians in western Europe generally have stronger role compare to those in the eastern Europe [5]. The practice among countries in eastern Europe itself shows considerable differences [6].

Our study shown that many of the Indonesian primary care physicians do not provide services usually carried-out by primary care physicians in other countries. With regard to the four areas of service provision provided by primary care as defined by Grielen et al., [6] we found that services provided by Indonesian PCP were especially lacking in the application of medical procedures such as minor surgical and investigative procedures, management and follows up of acute and chronic conditions, and preventive medicine and health education.

The low availability of certain investigative procedures such as respiratory function test, electrocardiography, ultrasonography, visual examination with an ophthalmoscope or proctoscopy might due to the cost of equipment and cost of services. When this survey was conducted, universal coverage had not been implemented in Indonesia thus higher proportion of Indonesian population was not covered by health insurance. Yet, it is possible to encourage the use of some equipment such as ophthalmoscope or simple respiratory function test which is quite affordable. Provision of those services determine the comprehensiveness of primary care services [10]; one of the role intended for primary care [11].

Non-communicable diseases including cardio-vascular diseases, diabetes mellitus, chronic respiratory problem, mental health problem and substance abuse are emerging as the major threat in Indonesia [12] and the PCPs were expected to be actively involved in managing those problems. Yet in this survey we found that low proportion of PCPs provide services that were highly relevant to those problems such as body mass index (BMI) measurement, cardiovascular assessment substance abuse program and mental health assessment.

It was found that Family Physicians tend to provide more house call services and less acupuncture and hypnotherapy compare to those who are not Family Physicians. Those who practice more than 30 hours per week tend to have facilities to cope with emergency care, providing substance abuse program, treadmill assessment, urine examination, blood glucose test, ECG, minor surgery and soft tissue infiltration, have separate register for chronic disease and electronic medical record when compared to those who practiced less than 30 hours a week.

This pilot study was limited in that it was conducted among a selected group of delegates who attended a conference. It is evident that this cross-sectional survey studied the primary care physicians who worked in the bigger cities of Indonesia and has shown that encouraging primary care physicians to submit to a conversion program has elevated the practitioners to a higher level of care in terms of skills and services in a developing country like Indonesia. Further this study has shown that longer hours of practice also improves provision of care by primary care practitioners possibly due to the higher number and variability of patients/cases managed by the physicians. As Roger Jones [13] argues, strengthening general practice especially with strong educational support is the basis of primary healthcare system and not secondary or tertiary care. Profiling general practice lends to assessing the current status in order to springboard methods of improving the system.

Differences between family physicians and non-family physicians were small with respect to range of services, facilities and continuing medical education. This was unexpected since conversion program was envisioned to recognize those already implement family medicine approach prior to structured Family Medicine training is made available. It appears that the checklist utilized in the conversion program failed to distinguish those who provide better range of services. Remedial action may be proposed including developing better instrument which better reflecting the area of services provided by PCPs followed by provision of structured trainings focusing on the essential services which are currently less provided.

With regard to the availability of formal postgraduate training program for primary care practice, Indonesia is still lagging compare to other member countries of the Association of South East Asian Nation (ASEAN) and the role of primary care is still weakly recognized [2,7]. With the national governments struggling to contain ever increasing health care costs, the gate keeper role (i.e. provision of first contact services) of primary care should be strengthened. Strengthening the gatekeeping function of the PCPs and implementation of referral system will improve the provision of comprehensive services [5,14].

This is also in line with the current a movement of the ASEAN countries through establishment of the ASEAN Region Primary Care Physicians Association which one of the aim is “to work towards common standards for quality healthcare, education, training, accreditation and certification to set competencies for general practitioners/family physicians” in the region. This study provide important information to support the movement.

## Conclusion

The Indonesian PCPs in our survey were lacking in the provision of some particular medical procedures, management and follows up of acute and chronic conditions, and preventive medicine and health education. However, longer hours of practice improves provision of services by primary care physicians more than participation in family medicine conversion program. Improvement of primary health care has been seen globally as necessary effort in health systems reform [15]. The results of our study show in which area the role of the Indonesian PCPs is relatively weak and on which skills the emphasis needs to be placed. This can provide guidance for the development of training programs for GPs to meet the common standards of the ASEAN countries.

## References

[1] Starfield B (1994). Is primary care essential?. Lancet.

[2] Hays R, Pong LT, Leopando Z (2012). Primary care in the Asia-Pacific region: challenges and solutions. Asia Pac Fam Med.

[3] Valderas J, Starfield B, Forrest C, Sibbald B, Roland M (2009). Ambulatory care provided by office-based specialists in the United States. Ann Fam Med.

[4] Hider P, Lay-Yee R, Crampton P, Davis P (2007). Comparison of services provided by urban commercial, community-governed and traditional primary care practices in New Zealand. J Health Serv Res Policy.

[5] Boerma W, van der Zee J, Fleming D (1997). Service profiles of general practitioners in Europe. European GP Task Profile Study. Br J Gen Pract.

[6] Grielen S, Boerma W, Groenewegen P (2000). Task profiles of general practitioners in Central and Eastern Europe. Eur J Pub Health.

[7] Hays RB, Morgan S (2011). Australian and overseas models of general practice training. Med J Aust.

[8] Wonodirekso S, Pattiradjawane D (2010). The Role of the Ministry of Health in Empowerment and Career Development of Primary Physician to Achieve “MDGs” Targets. J Indones Med Assoc.

[9] Academy of Family Physicians of Malaysia (2013). Quality Improvement Program.

[10] Kringos DS (2012). The strength of primary care in Europe.

[11] Starfield B (1998). Primary Care: balancing health needs, services, and technology.

[12] World Health Organization Regional Office for South-East Asia (2007). Country profile: Indonesia. 11 Health questions about the 11 SEAR countries.

[13] Jones R (2010). Strong medicine: research, education, and patient care in general practice. Br J Gen Pract.

[14] Boerma W, Verhaak P (1999). The general practitioner as the first contacted health professional by patients with psychosocial problems: a European study. Psychol Med.

[15] World Health Organization (2008). Primary health care now more than ever.

